# Mobile Apps for Health Behavior Change in Physical Activity, Diet, Drug and Alcohol Use, and Mental Health: Systematic Review

**DOI:** 10.2196/17046

**Published:** 2020-03-18

**Authors:** Madison Milne-Ives, Ching Lam, Caroline De Cock, Michelle Helena Van Velthoven, Edward Meinert

**Affiliations:** 1 Digitally Enabled Preventative Health Research Group Department of Paediatrics University of Oxford Oxford United Kingdom; 2 Department of Primary Care and Public Health Imperial College London London United Kingdom

**Keywords:** telemedicine, evidence-based medicine, mobile health, digital health, mobile applications, app, cell phone, smartphone, mobile phone, health behavior, intervention, behavior change, systematic review

## Abstract

**Background:**

With a growing focus on patient interaction with health management, mobile apps are increasingly used to deliver behavioral health interventions. The large variation in these mobile health apps—their target patient group, health behavior, and behavioral change strategies—has resulted in a large but incohesive body of literature.

**Objective:**

This systematic review aimed to assess the effectiveness of mobile apps in improving health behaviors and outcomes and to examine the inclusion and effectiveness of behavior change techniques (BCTs) in mobile health apps.

**Methods:**

PubMed, EMBASE, CINAHL, and Web of Science were systematically searched for articles published between 2014 and 2019 that evaluated mobile apps for health behavior change. Two authors independently screened and selected studies according to the eligibility criteria. Data were extracted and the risk of bias was assessed by one reviewer and validated by a second reviewer.

**Results:**

A total of 52 randomized controlled trials met the inclusion criteria and were included in the analysis—37 studies focused on physical activity, diet, or a combination of both, 11 on drug and alcohol use, and 4 on mental health. Participant perceptions were generally positive—only one app was rated as less helpful and satisfactory than the control—and the studies that measured engagement and usability found relatively high study completion rates (mean 83%; n=18, N=39) and ease-of-use ratings (3 significantly better than control, 9/15 rated >70%). However, there was little evidence of changed behavior or health outcomes.

**Conclusions:**

There was no strong evidence in support of the effectiveness of mobile apps in improving health behaviors or outcomes because few studies found significant differences between the app and control groups. Further research is needed to identify the BCTs that are most effective at promoting behavior change. Improved reporting is necessary to accurately evaluate the mobile health app effectiveness and risk of bias.

## Introduction

### Background

Engaging patients with health care is an important area of development in health care because it has the potential to reduce preventable deaths [1,2]. There is a huge range of digital health technologies that can deliver health care interventions, including apps, SMS texts, emails, internet, interactive chatbots, and voice agents [3-5]. Since the first iPhone was released in 2008, smartphone technology has become increasingly prevalent and capable, offering a promising means of delivering health care interventions to the general population. The large number of mobile health apps currently available for download is a testament to their popularity [6]. Many mobile phones now have the ability to passively collect a variety of health data—including physical activity, social interaction, sleep, and mobility patterns— and make inferences about mental and physical health [7,8]. Combining these capabilities with active user interaction allows mobile apps to deliver many different behavioral interventions, which can help users lead healthier lives and potentially reduce the likelihood of preventable health issues.

Mobile apps have been designed to target a wide variety of actions to prevent problems and maintain and improve patients’ health [9]. There are five main types of health behaviors—physical activity, diet, drug use, alcohol use, and mental health [4]—but other actions such as the management of chronic conditions, medication adherence, doctor appointments, vaccinations, dental hygiene, sun protection, and sex safety can also be considered health behaviors [10,11]. However, mobile health apps’ effectiveness has not been sufficiently established [4,12,13]. Many studies do not even report whether or not their mobile health behavior apps are based on behavioral theories. Although there is a debate on the role behavioral interventions can and should play in the population-level behavior, behavioral theory is agreed to be an important component of successful health-related behavioral interventions [14]. Further evaluation of the effectiveness of mobile health apps is needed to determine which apps are most useful and which behavioral change theories and techniques best promote positive behavior change, which in turn can guide future development. This is important because of the ubiquity of mobile health apps in society—if they are to fulfill their intended goal of improving health, they must be able to effectively improve and maintain positive health behaviors.

Many systematic reviews are currently examining these topics. However, with a few exceptions [12,15], most of these reviews are restricted in scope to specific types of health behaviors, patient groups, or combinations thereof. This has the advantage of being able to more directly compare the studies, and perform meta-analyses, if the studies are similar enough. The diversity of the mobile health app field makes it difficult to coalesce the results of studies into a coherent overview. A few systematic reviews have taken on this challenge. These examined studies focused on a variety of health behaviors, patient groups, or types of app intervention. Overall, they found that mobile health apps were effective in improving participants’ health behaviors [12,15]. They also identified the most commonly used behavior change constructs: self-monitoring [13,15], cues to action, feedback, and social support [15] However, each of these systematic reviews has limitations. These limitations include the exclusive use of broad search terms, which likely missed many relevant mobile health app articles that used more specific key terms [12], and that the articles reviewed were primarily pilot studies with small sample sizes [15]. In addition, data for these systemic reviews were collected in 2014 [15], 2015 [13], and 2017 [12]. Given the rapid pace of technological development, a new systematic review is necessary to provide an accurate assessment of the effectiveness of the most recent mobile health app interventions.

Identifying the behavior change techniques (BCTs) that are most effective in fostering positive change is necessary to develop the most effective and engaging interventions to improve the participant health behavior. An update to and expansion of previous systematic reviews is needed to provide an overview of current mobile health app technology, the BCTs being used, and their effectiveness in changing behavior and participant health outcomes. New, innovative apps are continuously being developed and tested, and systematic reviews must keep pace so that overall trends in the features, theories, and effectiveness of these apps can be tracked and updated. To ensure that the mobile health apps that patients are using are achieving their promises of health behavior change, it is essential to have a clear understanding of what is currently being used and whether it is working. If barriers—in the apps’ features or use—can be identified, app developers can use that information to design more effective interventions.

### Objectives

The primary objectives of the review are to summarize the state of the field of mobile health apps for behavior change and evaluate their effectiveness. The wide variety of apps and health behaviors examined means that there are a wide range of outcomes examined to address three main research questions. First, what types of mobile health apps and BCTs are being used to support user engagement with their health behaviors? Second, how effective are mobile health apps in improving and maintaining positive health behavior changes? Finally, what are participant perceptions of the feasibility, functionality, and overall user experience of the mobile health apps they use? These are the key elements that are needed to comprehensively evaluate mobile health apps. This focus builds on previous systematic reviews and extends and updates the body of knowledge on current mobile health apps to inform further research and development.

## Methods

### Database Search

The methods are described in detail in a systematic review protocol that is registered with the International Prospective Register of Systematic Reviews (PROSPERO: CRD42019155604). The search strategy was developed using the Population, Intervention, Comparison, and Outcome (PICO) template and performed following the Preferred Reporting Items for Systematic Reviews and Meta-Analyses Protocols (PRISMA-P Multimedia Appendix 1) [16]. MeSH terms and keywords were extracted from a preliminary review of the literature, and the search strings and databases were decided in consultation with a medical librarian. The search was performed in four databases using the University of Oxford Search Oxford Libraries Online—PubMed, EMBASE, Cumulative Index to Nursing and Allied Health Literature, and Web of Science—with slightly adjusted search terms to fit the specific structure of each database. The search terms were grouped into four themes—mobile phones, mobile apps, health behaviors, and evaluation—that were joined with the structure: Mobile (MeSH OR Keywords) AND Applications (MeSH OR Keywords) AND Health Behavior (MeSH OR Keywords) AND Evaluation (MeSH OR Keywords). A complete list of the specific search terms and strings used for each database is provided in Multimedia Appendices 2 and 3. The search was completed on September 16, 2019.

### Inclusion and Exclusion Criteria

Digital health technologies evolve rapidly, and this review was concerned with the current state of mobile health app technology [17]. Therefore, the search was limited to studies published between 2014 and 2019. This time frame provided an update to Payne et al’s [15] systematic review that included studies published between 2007 and September 2014 and reflected the most recent behavioral and technological developments. Only studies published in English were included.

This systematic review had a broad scope for population and included any age, gender, country, or ethnicity. Therefore, study populations could be general or specific with regard to demographic variables. However, to keep the focus on the effectiveness of mobile health apps in the general population, subgroups such as pregnant women and patients with specific diseases (including HIV, posttraumatic stress disorder, alcoholism, and chronic depression) were excluded.

The intervention targeted in this review was mobile apps for health behavior change. Therefore, to be included, the main focus of the study needed to be the evaluation of a mobile app that helps users adopt, improve, or maintain positive health behaviors. Studies of mobile interventions that did not evaluate the app, were designed for use by health care professionals, focused on behavior change theory without reference to mobile apps, or focused on mobile phones or wearable technology but not apps—for instance, interventions based solely on SMS text messaging or emails—were excluded. Interventions that were primarily focused on mobile apps but involved wearable technology for data collection were included.

Initially, we intended to include all types of health behaviors. However, the number of studies that this would have entailed was unfeasible, and the health behaviors that were included in the final systematic review were limited to the five main categories established in the literature: drug use, alcohol use, diet, physical activity, and mental health. This excluded behaviors such as sun protection, sex safety, medication adherence, doctor’s appointments, vaccinations, and self-management of chronic conditions.

Study design was not limited in the initial search to ensure that no relevant studies were missed, but only randomized controlled trials were included in the review. All types of comparators were included.

### Outcomes Measured

The primary outcomes were participant health and behavior change to evaluate the apps’ effectiveness. Secondary outcomes included the apps’ features and their adoption of specific BCTs [18], as well as engagement and adherence rates, participant-reported experience, and feasibility and usability assessments.

### Screening and Study Selection

All the articles identified from the database searches were stored in the citation management software to eliminate duplicates before the abstracts were screened by two independent reviewers. Disagreements were discussed until consensus on eligibility was reached. The full text was screened by one of the reviewers, and when the text did not meet the inclusion criteria, the second reviewer reviewed the article to assess eligibility to determine inclusion in the final set. Reasons for inclusion and exclusion were recorded at both the abstract and full-text screening stages.

To check if the search had missed any relevant articles, the full citation list was compared with the list of studies included in the two previous systematic reviews [12,15]. Of the 20 articles examined in Han and Lee’s [12] review, 11 were already included in the citation list, and none of the other 9 were eligible for inclusion [12]. As Payne et al’s [15] review finished data collection in 2014, only 8 of their 24 studies were within the time frame of this review [15]. Of those, 6 were already in the citation list, 1 had just been identified from the Han and Lee’s review [12], and the other was added to the overall citation list but excluded from the final review because it was a treatment for major depressive disorder.

A total of 8 of the screened articles eligible for inclusion were abstracts from posters, conferences, or meetings and did not have full texts available. The authors of each were contacted to request a full text if it was available. At the time of writing, only one of the study authors had replied. A full text was sent but ended up not being relevant and was excluded from the final review because a mobile app was not the main focus of the study.

### Data Extraction

Data were extracted by one reviewer and key data points from the studies that were specified in the protocol were recorded in a spreadsheet (see Table 1). This process was validated by the second reviewer, and disagreements were discussed with a third reviewer. The broad scope of the review meant that there were a wide variety of specific health and behavior change outcomes, so a meta-analysis could not be performed.

**Table 1:** Data that were extracted from the studies.

Article information	Data extracted
General study information	Year of publication Countries of study Study setting (primary location of app use, if relevant) Analyzed sample size Sample demographics (including age, gender, and target population) Intervention duration and follow-up periods
Behavioral intervention	Target health behaviors and intervention focus Theory the intervention is based on Behavior change techniques (BCT Taxonomy v1 [18,19])
Mobile app technology	Area of health care used in Name of the app Developers Platform Components and design features (eg, provision of feedback, notifications, and tracking)
Evaluation	What outcomes were measured Participant health outcomes Behavior change outcomes Participant engagement or adherence rates Participant satisfaction Feasibility and usability Other key performance indicators reported

### Risk of Bias Assessment

The study design was limited to randomized controlled trials, so we used the Cochrane Collaboration Risk of Bias tool to assess all of the included articles [20]. Specifically, this assessed the risk of bias in random sequence generation; allocation concealment; blinding of participants, personnel, and outcome assessors; incomplete outcome data; and selective outcome reporting. The risk of bias assessment was conducted by one reviewer and validated by the second reviewer, and disagreements were resolved by discussion.

### Data Analysis and Synthesis

The variety of study aims, methods, and reported outcomes meant that a meta-analysis was unfeasible, so a narrative summary of the studies was prepared to draw conclusions about the apps’ effectiveness, use of BCTs, acceptability, and usability. In this review, an outcome was only considered to have significant evidence supporting it if the app performed significantly better than a comparator or control. Outcomes that were significantly different over time but not between groups were coded as having some evidence supporting them. Outcomes that were not significantly different between groups, had no significant effect, or were significantly worse than the comparator were coded as having no evidence supporting them. Limitations and future directions for research and development were also identified.

## Results

### Included Studies

**Figure 1:**
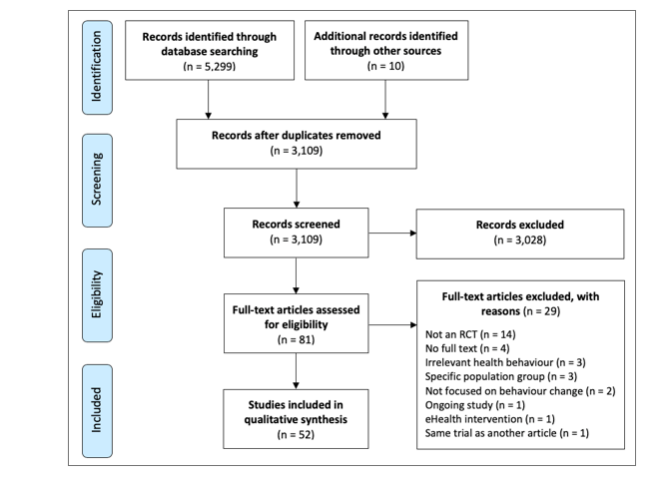
Preferred Reporting Items for Systematic Reviews and Meta-Analyses flow diagram. eHealth: electronic health; RCT: randomized controlled trial.

In total, we retrieved 5299 articles using the search terms in the four databases. Of these, 81 were selected for the full-text review, and 52 were selected for inclusion in the review. The reasons for exclusion in the full-text review stage are detailed in Figure 1. One article was excluded at the full-text stage for reporting the same trial as another article that was already included. This excluded article was more focused on the development of the app and did not provide any additional relevant data for extraction.

### Study Characteristics

The characteristics of the 52 included studies are summarized in Multimedia Appendix 4. Of these 52, 71% (n=37) aimed to change dietary habits, physical activity, or both to reduce or prevent obesity and improve general health. The goal of 21% (11/52) of the studies was to reduce drug and alcohol use (9/52 were related to smoking cessation, and 2/52 aimed to reduce alcohol consumption). The last 8% (4/52) targeted behaviors aimed at improving mental health. More than half of the studies (28/52) had sample sizes of less than 100 participants, and slightly fewer than half (23/52) had a study duration less than 3 months.

### Overall Effectiveness of Apps

One study was excluded from this part of the analysis because it only evaluated feasibility, usability, and participant perceptions [21]. Only about a quarter of the studies (12/51) found that the app had a significantly better effect (*P*<.05) on participant health or behavior change outcomes than control or comparator groups (see Table 2). These include significantly bigger increases for the app group in healthy food consumption (eg, 1-2 more daily servings of vegetables [22,23]), physical activity (eg, 1000-2000 more steps per day [24,25]), and mental health (large Cohen *d* effect sizes for mindfulness and self-compassion , 0.8 and 1.1, respectively [26]), as well as significantly bigger decreases in drug and alcohol use [27,28]. About 30% of the studies (16/51) found some evidence of effectiveness—whether there was a significant difference between the groups on some but not all of the outcomes, a significant difference over time but not between groups, or a significant improvement over the control only in a subgroup of the population. The remaining 45% of studies either found no significant difference between groups or effect on the primary outcome (22/51) or found that the app performed worse than the comparator (1/51). This analysis was done by coding the overall outcomes of each study, but the ratio is very similar if the overall effectiveness is coded once for each individual app instead.

**Table 2:** Summary of general evidence of effectiveness by study (N=51).

Evidence of effectivenes?	Physical activity (PA) [24,25,29-39], n	Diet [22,23,40-49], n	Diet and PA^a^ [50-60], n	Mental health [26,61-63], n	Smoking cessation [28,64-71], n	Reduce alcohol [27,72], n	Total, n (%)
No	4	3	7	1	7	1	23 (45)
Some	4	5	4	2	1	0	16 (31)
Yes	5	4^b^	0	1	1	1	12 (23)
Total	13	12	11	4	9	2	51 (100)

^a^The studies in the diet and physical activity category reported on dietary and physical activity outcomes, whereas the studies in the previous 2 columns reported on either diet or physical activity.

^b^Two of these studies report on the same trial (one at 12 weeks and the other at the end of the 12-month trial) [22,47]. Both have been included in this table, but if one were excluded, there would be significant evidence for 22% (11/50) studies.

#### Participant Health Outcomes

A wide range of participant health outcomes were reported in 20 studies, including measures of weight change over time (weight, BMI, waist circumference, and body adiposity), mental well-being (depression, anxiety, life satisfaction, perceived stress, emotional regulation, etc), blood pressure, cardiovascular risk factors, and biomarkers such as blood lipids and urinary sodium. Overall, there was very little evidence that supported the effectiveness of mobile health apps to affect participant health outcomes (see Table 3). Over three-quarters (24/31) of the reported participant health outcomes were not significantly different between the intervention and control groups.

**Table 3:** Effectiveness of apps on participant health outcomes (N=31).

Participant health outcome	No evidence, n	Some evidence, n	Significant evidence, n (%)	Studies reporting outcome, n
Weight/BMI change [35,41-46,48,50,55,59,60]	10	1	1 (8)	12
Waist circumference/body adiposity [43,53,59]	2	1	0 (0)	3
Mental well-being (eg, depression, anxiety, perceived stress, life satisfaction, and mood) [26,50,61-63]	2	3	0 (0)	5
Blood pressure [35,41,46,51,54]	5	0	0 (0)	5
Cardiovascular risk factors [54,55]	2	0	0 (0)	2
Blood measures (eg, blood glucose and blood lipids) [41,54]	2	0	0 (0)	2
Urinary sodium [43,51]	1	1	0 (0)	2
Total	24	6	1 (3)	31

#### Behavior Change Outcomes

An even broader range of behavior change outcomes were reported in 44 of the 52 studies. Overall, there was not much significant evidence supporting the effectiveness of apps in changing the behavior outcomes (see Table 4). There were certain types of behavior that had stronger evidence of change than others. A total of 63% (5/8) of studies that examined healthy food choice behavior found that the app group improved significantly more than the control or comparator group, as did 43% (3/7) of the studies that reported step count and 100% (3/3) of the studies that aimed to reduce sedentary behavior. Physical activity and dietary habits were the target behavior areas with the highest percentage of significant evidence (32% each). However, there were only a few studies for each specific outcome, and altogether, the studies only found significant support for the effectiveness of mobile health apps in just a quarter (16/64) of the behavior change outcomes reported.

**Table 4:** Effectiveness of apps with respect to behavior change outcomes (N=44).

Target behavior and behavior change outcome^a^		No evidence, n	Some evidence, n	Significant evidence, n (%)	Total times outcome reported, n
**Dietary habits**					
	Healthy food choices (including vegetable consumption and purchase of salt) [22,23,40,44,45,48,51,59]	2	1	5 (63)	8
	Hunger [40]	1	0	0 (0)	1
	Control (including cognitive restraint, self-efficacy, self-regulation, PBC^b^, and avoiding uncontrolled eating) [40,41,45,46,49,60]	5	0	1 (17)	6
	Dietary compliance (including goal setting and diet tracking) [42,45,46,52,58]	4	0	1 (20)	5
	Energy/caloric intake [55,57]	1	1	0 (0)	2
	Total (dietary habits)	13	2	7 (32)	22
**Physical activity**					
	Physical activity (including moderate to vigorous physical activity) [25,30,33,37-39,52,55,58]	7	1	1 (11)	9
	Walking/step count [24,25,29,33,35,57]	2	2	3 (43)	7
	Reduce sedentary behavior [29,32,37]	0	0	3 (100)	3
	Time to complete fitness test [31]	1	0	0 (0)	1
	Attitudes to physical activity [34]	0	1	0 (0)	1
	Control (including self-efficacy, PBC, and barriers) [25,34,39]	2	1	0 (0)	3
	Self-monitoring [39]	0	0	1 (100)	1
	Total (physical activity)	12	5	8 (32)	25
**Reduce alcohol**					
	Change in weekly alcohol consumption [27]	1	0	0 (0)	1
	Change in full Alcohol Use Disorders Identification Test score [27]	1	0	0 (0)	1
	Number of alcohol consequences [72]	1	0	0 (0)	1
	Maximum number of drinks at once [72]	1	0	0 (0)	1
	Total (reduce alcohol)	4	0	0 (0)	4
**Smoking cessation**					
	Continuous abstinence (including 7- and 30-day point prevalence abstinence) [28,64,67-71]	5	1	1 (14)	7
	Quit rates [65,66]	2	0	0 (0)	2
	Acceptance of cravings [65]	0	1	0 (0)	1
	Readiness to quit (including motivation and quit attempts) [66,69,71]	3	0	0 (0)	3
	Total (smoking cessation)	10	2	1 (8)	13
Total		39	9	16 (25)	64

^a^Many of the studies reported more than one behavior change outcome, and all distinct outcomes were recorded here, so there are more individual outcomes than the number of studies.

^b^PBC: perceived behavioral control.

### Behavior Change Techniques and Theories

In the 52 studies, there were 50 unique apps tested (excluding basic or control versions of apps). Only a few of these studies explicitly reported the BCTs incorporated into the app, so BCTs were coded based on the study descriptions of the app features using the BCT Taxonomy v1 [18]. The taxonomy lists 93 BCTs, clustered into 16 groups. Collectively, the apps studied included 39 different BCTs from 12 different groups. Multimedia Appendix 5 reports the BCTs included in each app studied. Only four BCTs were used in more than half of the apps—1.1 Goal setting (behavior; 52% of apps), 2.2 Feedback on behavior (54%), 2.3 Self-monitoring of behavior (72%), and 4.1 Instructions on how to perform the behavior (54%). The mean (and median) number of BCTs per app was 5 (range: 0-11), and the most common number of BCTs per app was 6.

An exploratory assessment of the effectiveness of each BCT was conducted by associating the use of BCTs in each app with the effectiveness of that app, so that a count of how many times the BCT was associated with significant evidence versus no evidence could be made. There was at best mixed evidence for all of the BCTs used. Only 4 BCTs (1.6 Discrepancy between current behavior and goal, 4.2 Information about antecedents, 6.1 Demonstration of the behavior, and 12.5 Adding objects to the environment) had more significant evidence than not, but only by 1 study. These 4 BCTs were also only used in at most 2 apps. The most frequently used BCTs were all associated two to three times more with studies that found no significant effect compared with those that found a significant effect on the specified outcomes.

Half of the studies (26/52) mentioned the specific behavioral theories that were considered when developing the app, and there was a lot of variety. A total of 23 different theories were referenced, with social cognitive theory and behavior change theory being referenced most frequently (8 and 5 times, respectively), with the remaining 21 theories having no more than two mentions each. Only 5 of these 26 studies found significant evidence in favor of the app. Of these 5, 2 used social cognitive theory, 2 used bahavior change theory, and 1 used the capability, opportunity, motivation, behavior framework and the behavior change wheel.

### Engagement and Adherence

Engagement and adherence outcomes were reported by 39 of the studies. Of these, 18 reported completion or retention rates, and 26 reported the app use data. The mean completion rate across studies was 83.3% (range: 45%-97.1%), with 8 studies reporting a completion rate above 90%. There was significant variability in what app use measures were reported and how they were used to evaluate adherence. A total of 4 studies reported that the app group was significantly more engaged with their intervention than the control group, and 2 more reported high use in the app group but not whether the difference was significant. A total of 9 studies reported a usage percentage greater than 60%.

### Feasibility and Usability

A total of 15 studies reported on usability (n=13), feasibility (n=1), or both (n=1). A total of 3 of these studies reported that the intervention app was rated significantly better than the control, and 9 more reported high ease of use (>70% of participants rated highly). There does not appear to be any relationship between usability and effectiveness, given the generally high usability ratings and overall low effectiveness. However, as less than a third of studies reported usability, this analysis should be treated with caution.

### Participant Satisfaction

A total of 21 studies reported participant satisfaction. Of these studies, 2 reported significantly higher ratings for the intervention app than the control, and 6 more reported that more than 70% of the participants rated the app as satisfying, helpful, or enjoyable. Only 1 app (Crush the Crave) was found to have significantly lower helpfulness and satisfaction ratings than the control. The rest had mixed feedback or no significant differences between the ratings for the app and control.

### Risk of Bias Assessment

**Figure 2:**
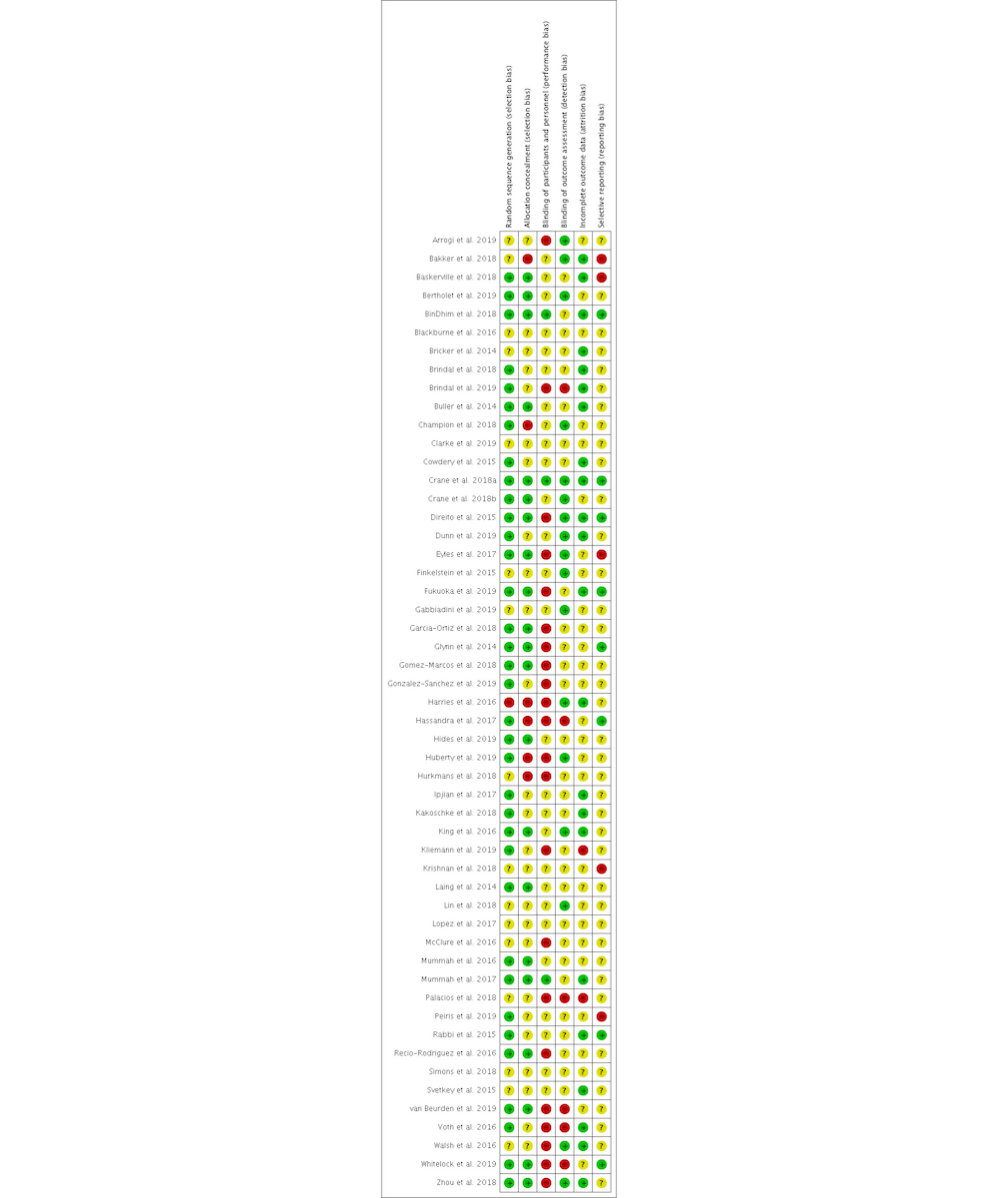
Risk of bias summary: the review authors’ judgements about each risk of bias item for each included study.

**Figure 3:**
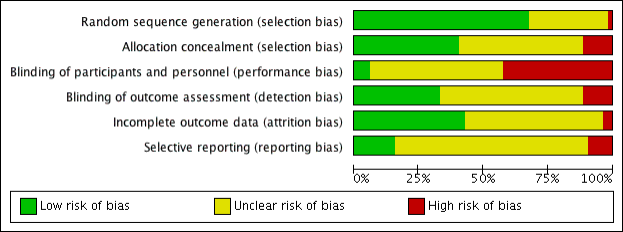
Risk of bias graph: the review authors’ judgements about each risk of bias item presented as percentages across all included studies.

The evaluation of risk of bias for all 52 studies was conducted using the Cochrane Collaboration Risk of Bias tool [20], and the results were summarized using the RevMan 5.3 software (Figures 2 and 3) [73]. Two-thirds of the studies (35/52) properly reported random sequence generation [36].

About 40% (21/52) of the studies reported satisfactory allocation concealment and either very low attrition or no significant differences in attrition between groups (22/52), meaning that the risk of incomplete outcome data was low. About a third of studies (17/52) reported blinding of outcome assessors, but only 3 studies reported blinding of patients and personnel. This was predominantly because the nature of mobile app interventions made blinding of participants difficult. A total of 42% (22/52) of the studies had a high risk for the blinding of participants and personnel, predominantly because they reported that blinding was not possible. However, only 15% (8/52) of the studies could be established as having a low risk of selective outcome, mostly because a preregistration or study protocol could not be found to compare reported outcomes with.

## Discussion

### Principal Findings

Developments in technology have made it easier for patients to play an active role in their health, and there are thousands of mobile apps designed to help people improve their health behaviors [4]. However, their effectiveness in changing health behaviors and outcomes has not been convincingly established. In this systematic review, we examined 52 randomized controlled trials evaluating the effectiveness of mobile health apps. Overall, there was little significant evidence supporting the effectiveness of mobile health apps for any of those outcomes. This was especially true for patient health outcomes; out of all the studies that examined them, there was only 1 app that was significantly better than the control. There was slightly more evidence for the effectiveness of apps in changing the health behavior outcomes—apps performed significantly better than controls on a quarter of the measured outcomes—but a majority of outcomes reported no significant differences between groups.

This is a different finding than previous systematic reviews. Both Han and Lee [12] and Payne et al [15] reported that the majority of apps reviewed were effective at improving health-related behaviors. It is possible that this difference is because of the way study results were interpreted. Only considering studies that found a significant difference between groups in favor of the app as significant evidence of effectiveness is a conservative interpretation. However, randomized controlled trials are the gold standard for evaluating interventions because randomization allows differences in outcomes between groups to be attributed to the intervention [74]. To conclusively support the claim that mobile health apps are a useful tool for changing behavior and health outcomes, users should show greater improvement with the app than a comparator or control. The majority of the apps evaluated in this review were not significantly more effective in achieving their purposes than controls or alternative interventions.

When significant and some evidence categories are both taken into account, there was moderately strong evidence supporting the effectiveness of apps in changing certain target behaviors, notably, healthy food choices (6/8, 75%), step count (5/7, 71%), and reducing sedentary behavior (3/3, 100%). This suggests that mobile apps do have the potential to improve some health behaviors. However, the number of studies that examined each of these outcomes was small. In addition, this effect was not reflected in any of the participant health outcomes. Possibly the intervention durations were too short for any meaningful clinical change, although half of the studies lasted longer than 3 months, and modest weight loss (5%-10%) can be observed within 3 to 6 months [75]. Even when the behavior change was significantly greater in the app than the control group, it may not have been enough of a change to induce an observable effect in any of the health outcomes measured over time.

Before delving too deeply into clinical outcomes, however, it is first essential to have a measurable behavioral effect. Identifying the BCTs that are most effective in promoting and maintaining positive health behavior change is crucial for the development of mobile apps that will significantly improve health behaviors and outcomes [76]. However, determining which BCTs, and combinations of BCTs, are most effective in specific contexts is a complex process, and a valid method of determining the degree of confidence of BCT effectiveness is yet to be established [76]. To make this even more difficult, most of the studies did not report the BCTs used, and they had to be inferred from the descriptions of the apps’ features. Self-monitoring of behavior was the most commonly used BCT (72% of apps included a self-monitoring function). Behavioral goal setting, feedback on behavior, and instructions on how to perform the behavior were also included in more than half of the apps. This is consistent with the findings of Payne et al’s [15] systematic review of mobile health apps, which found that most studies included goal setting, self-monitoring, and social support constructs. Social support was not as prominent in the apps studied in this review, with only 28% having an identifiable social support feature. If there is a disconnect between the BCTs that are most effective and those that are most frequently used, it could explain the lack of behavioral change.

For all 4 of the most frequently used BCTs, there were far more studies with no evidence than significant evidence, with less than a fifth of the studies of apps using those individual BCTs finding a significant effect (range: 15%-19% per BCT). This is similar to the low overall amount of significant evidence supporting the apps’ effectiveness, which is not surprising. Self-monitoring has been positively associated with behavior change in the literature, though the results are heterogenous [77,78]. Therefore, why are these studies not finding much support for their effectiveness in changing behavior?

Intuitively, a greater number of BCTs might seem more likely to improve health behaviors, or at least provide a wider range of motivating options so that users can choose the ones that work best for them. However, there was a similar average number of BCTs per app for each of the evidence groups (no, some, and significant evidence). The no evidence group actually had a higher average number of BCTs per app (5.9 vs 4.4 in the other two groups) but two-tailed *t* tests showed that none of the differences between the groups were significant (*P*>.05). This lack of an association between the number of BCTs and effectiveness is consistent with previous studies [79].

It is possible that the apps were not appropriately implementing the BCTs in their features. The lack of theoretical bases for half of the apps suggests that BCTs were not considered in many of them. However, only 4 of the 26 studies that did reference behavioral theories (actually 5 out of 27 studies, but 2 studies reported the same experiment [22,47]) found significant evidence for the app they studied, which is no better than the overall group of studies. This suggests that the inclusion of theory is not sufficient to find an effective result. The variety of theories used could also be a factor; further research should determine which theoretical bases are most strongly linked to behavior change. A more in-depth evaluation of the use and effectiveness of BCTs—as individual factors and in combination—in mobile health apps is necessary to understand why the majority of studies are not finding significant behavioral or health advantages for mobile app interventions. This is especially important because of the ubiquity of health apps that are available and being used and the urgent public health need to improve health behaviors to address increasing health care costs and improve healthy aging [80].

### Quality of the Evidence

After analyzing the risk of bias of the included studies, only the category of performance bias (blinding of participants and personnel) had a generally high risk of bias (22/52, 42%). Overall, however, just over half of the potential areas for bias had an unclear risk. Therefore, to improve the quality of studies and to make bias assessments more clear and useful, researchers should improve reporting of their methods, so that the risk of bias can be assessed more accurately. Out of all 52 studies, only 3 had 5 or more areas of bias categorized as low risk [27]. High-quality studies are needed to make a valid evaluation of the effectiveness of apps, so that there is less risk of poor study methodologies confusing the conclusions.

### Limitations

One limitation of this review is that a meta-analysis could not be conducted because of the heterogeneity of the studies and their reported outcomes. However, a proper meta-analysis would make the effectiveness of mobile health apps easier to determine and quantify. Another limitation is that the focus was limited to just five health behaviors. There are many mobile health apps designed to help patients manage chronic conditions such as diabetes, depression, and asthma. Therefore, the results of this review cannot be generalized to all health behaviors. In addition, this review only considered published randomized controlled trials. This may have missed more recently developed apps that have not progressed to that stage of testing yet, or that might not have been tested in an academic context. It may also overrepresent studies where an effect was found, as the grey literature was not searched for studies that may have found null results and not been published.

### Future Directions

An important future direction for research—and app development—is to examine more closely the theoretical basis of mobile health apps, which BCTs they are using, and how those BCTs are implemented. This is a crucial element in determining why mobile health apps are not consistently succeeding in improving health behaviors. If the most effective BCTs, and combinations thereof, can be identified, mobile health apps have the potential to advance preventive health care globally. Once consistent and effective means of motivating behavior change have been identified, the relationship between health behaviors and health outcomes should be reassessed and, if necessary, improved. To complement this, it is important for researchers to improve their reporting, so that the risk of bias of studies can be accurately assessed and only high-quality studies can be included in analyses.

### Conclusions

The purpose of this systematic review was to examine the effectiveness of mobile apps to improve health behaviors and outcomes and the inclusion and effectiveness of BCTs. Although apps generally had relatively high engagement, usability ratings, and user acceptability and satisfaction, the significance of evidence for delivering behavior change outcomes assessed was nominal. This study built on previous systematic reviews to provide an updated and comprehensive examination of current mobile health apps for the general population. It extended the literature by examining the relationship between BCTs and app effectiveness. In addition, this systematic review evaluated effectiveness more stringently than previous reviews to provide a balanced perspective on current app effectiveness and identify areas for improvement. Further research is needed to identify the behavior change theories and specific BCTs best suited to promote and maintain positive health behavior change through mobile app interventions. A reliable method of analyzing BCT effectiveness and more experiments comparing how behavior change outcomes differ depending on the combinations of BCTs used would be a useful next step. Given the inconsistent results of studies of mobile health app effectiveness, a greater integration of theory into app development and comparative examination of theories and BCTs in those apps will help drive innovation and the creation of more effective mobile health apps.

## Appendix

Multimedia Appendix 1 Preferred Reporting Items for Systematic Reviews and Meta-Analyses checklist.

Multimedia Appendix 2 Search Terms.

Multimedia Appendix 3 Search queries and the number of results for each database.

Multimedia Appendix 4 Summary of study characteristics.

Multimedia Appendix 5 Behaviour Change Techniques used in the apps studied.

## Abbreviations

BCT: behavior change technique
