# Supplementary material for: Mobile Apps for Health Behavior Change in Physical Activity, Diet, Drug and Alcohol Use, and Mental Health: Systematic Review
Source: JMIR Mhealth Uhealth. 2020 Mar 18;8(3):e17046. doi: 10.2196/17046 (PMC7113799; doi:10.2196/17046)
Supplement: Multimedia Appendix 2 [file mhealth_v8i3e17046_app2.docx]

Table 1. Search Terms

| **Category** | **MeSH** | **Keywords (in title or abstract)** |
| --- | --- | --- |
|  |  |  |
| Mobile | Cell Phone OR Telemedicine | Smartphone OR “mobile phone” OR “mHealth” OR “mobile health” |
| Applications | Mobile Applications | App OR apps OR “mobile app*” OR “smartphone app*” |
| Health Behaviour | Health Behavior OR Health Promotion OR Exercise OR Weight Loss OR Obesity (diet therapy, prevention & control, rehabilitation, therapy) OR Nutrition Therapy OR Diet OR Smoking Cessation OR Smoking Reduction OR Tobacco Use Cessation OR Alcohol Drinking (prevention & control, therapy) OR Mental Health OR Safe Sex OR Behavioral Medicine OR Chronic Disease | - “Health behaviour” OR “health behavior” OR “behaviour change” OR “behavior change” OR - (Exercise ADJ3 (increase or start or maintain*)) OR “physical activity” OR - “Weight loss” OR “healthy weight” OR “five a day” OR “diet” OR “nutrition” OR - ((Maintenance OR maintain* OR achiev* Or retain*) ADJ4 (weight goal OR “weight loss” OR “goal weight” OR BMI)) OR - (Smoking ADJ4 (cessation OR stop* OR quit* OR reduc*)) OR - (Alcohol ADJ4 (reduc* OR limit* OR decreas* OR “cutting down” OR “cut down” OR “cut back” OR less* OR curb* OR abstain OR “dry January”)) OR - “Protection from sun” OR “sun protection” OR “sun safe*” OR - ((Sex OR “sex* behaviour” OR “sex* behavior”) ADJ4 (safe* OR protect*)) OR - ((Alzheimer* disease OR arthritis OR asthma OR cancer OR COPD OR Crohn* disease OR cystic fibrosis OR dementia OR diabetes OR epilepsy OR heart disease OR HIV OR AIDS OR mood disorders OR bipolar OR depression OR anxiety OR multiple sclerosis OR Parkinson* disease) ADJ4 (manag* OR “self help” OR “self manag*” OR coping OR cope)) OR - “Health management” |
| Evaluation | Outcome Assessment (Health Care) | Feasibility OR usability OR “evaluat*” OR “outcome*” OR acceptability OR adherence OR “effectiv*” OR “adoption” OR “assess*” |
