# Supplementary material for: Mobile Apps for Health Behavior Change in Physical Activity, Diet, Drug and Alcohol Use, and Mental Health: Systematic Review
Source: JMIR Mhealth Uhealth. 2020 Mar 18;8(3):e17046. doi: 10.2196/17046 (PMC7113799; doi:10.2196/17046)
Supplement: Multimedia Appendix 3 [file mhealth_v8i3e17046_app3.docx]

Table 2. Search queries and number of results for each database

| **Database** | **Search query** | **Results** |
| --- | --- | --- |
|  |  |  |
| MEDLINE (Ovid) | (Cell Phone/ OR Telemedicine/ OR smartphone.ti,ab OR “mobile phone”.ti,ab OR (“mHealth” OR “mobile health”).ti,ab) **AND** (Mobile Applications/ OR (app OR apps OR “mobile app*” OR “smartphone app*”).ti,ab) **AND** (“Outcome Assessment (Health Care)”/ OR (feasibility OR usability OR “evaluat*” OR “outcome*” OR acceptability OR adherence OR “effectiv*” OR “adoption” OR “assess*”).ti,ab) **AND** (Health Behavior/ OR (“health behaviour” OR “health behavior”).ti,ab OR Health Promotion/ OR (“behaviour change” OR behavior change”).ti,ab OR Exercise/ OR Weight Loss/ (exercise ADJ3 (increase OR start OR maintain)).ti,ab OR “physical activity”.ti,ab OR (“weight loss” OR “healthy weight” OR “five a day” OR “diet” OR “nutrition”).ti,ab  OR ((Maintenance OR maintain* OR achiev* Or retain*) ADJ4 (weight goal OR “weight loss” OR “goal weight” OR BMI)).ti,ab OR Obesity/dh, pc, rh, th OR Nutrition Therapy/ OR Diet/ OR (Smoking ADJ4 (cessation OR stop* OR quit* OR reduc*)).ti,ab smoking cessation/ OR smoking reduction/ OR “tobacco use cessation”/ OR (Alcohol ADJ4 (reduc* OR limit* OR decreas* OR “cutting down” OR “cut down” OR “cut back” OR less* OR curb* OR abstain OR “dry January”)).ti,ab OR Alcohol Drinking/pc, th OR Mental Health/ OR (“Protection from sun” OR “sun protection” OR “sun safe*”).ti,ab OR Safe Sex/ OR ((Sex OR “sex* behaviour” OR “sex* behavior”) ADJ4 (safe* OR protect*)).ti,ab OR Behavioral Medicine/ OR Chronic Disease/dh, pc, th OR ((Alzheimer* disease OR arthritis OR asthma OR cancer OR COPD OR Crohn* disease OR cystic fibrosis OR dementia OR diabetes OR epilepsy OR heart disease OR HIV OR AIDS OR mood disorders OR bipolar OR depression OR anxiety OR multiple sclerosis OR Parkinson* disease) ADJ4 (manag* OR “self help” OR “self manag*” OR coping OR cope)).ti,ab OR “health management”.ti,ab | 1311 |
| Embase (Ovid)* | (mobile phone/ OR Telemedicine/ OR smartphone.ti,ab OR “mobile phone”.ti,ab OR (“mHealth” OR “mobile health”).ti,ab) **AND** (Mobile Application/ OR (app OR apps OR “mobile app*” OR “smartphone app*”).ti,ab) **AND** (Outcome Assessment/ OR (feasibility OR usability OR “evaluat*” OR “outcome*” OR acceptability OR adherence OR “effectiv*” OR “adoption” OR “assess*”).ti,ab) **AND** (Health Behavior/ OR (“health behaviour” OR “health behavior”).ti,ab OR Health Promotion/ OR (“behaviour change” OR behavior change”).ti,ab OR Exercise/ OR body weight loss/ (exercise ADJ3 (increase OR start OR maintain)).ti,ab OR “physical activity”.ti,ab OR (“weight loss” OR “healthy weight” OR “five a day” OR “diet” OR “nutrition”).ti,ab  OR ((Maintenance OR maintain* OR achiev* Or retain*) ADJ4 (weight goal OR “weight loss” OR “goal weight” OR BMI)).ti,ab OR Obesity/dm, pc, rh, th OR diet therapy/ OR Diet/ OR (Smoking ADJ4 (cessation OR stop* OR quit* OR reduc*)).ti,ab smoking cessation/ OR smoking reduction/ OR “tobacco use cessation”/ OR (Alcohol ADJ4 (reduc* OR limit* OR decreas* OR “cutting down” OR “cut down” OR “cut back” OR less* OR curb* OR abstain OR “dry January”)).ti,ab OR drinking behaviour/pc, th OR Mental Health/ OR (“Protection from sun” OR “sun protection” OR “sun safe*”).ti,ab OR Safe Sex/ OR ((Sex OR “sex* behaviour” OR “sex* behavior”) ADJ4 (safe* OR protect*)).ti,ab OR Behavioral Medicine/ OR Chronic Disease/dm, pc, th OR ((Alzheimer* disease OR arthritis OR asthma OR cancer OR COPD OR Crohn* disease OR cystic fibrosis OR dementia OR diabetes OR epilepsy OR heart disease OR HIV OR AIDS OR mood disorders OR bipolar OR depression OR anxiety OR multiple sclerosis OR Parkinson* disease) ADJ4 (manag* OR “self help” OR “self manag*” OR coping OR cope)).ti,ab OR “health management”.ti,ab | 1630 |
| CINAHL (EBSCO)^a^ | ((MH "Cellular Phone") OR (MH "Telemedicine") OR (TI ( Smartphone OR “mobile phone” OR “mHealth” OR “mobile health”) OR AB ( Smartphone OR “mobile phone” OR “mHealth” OR “mobile health”))) **AND** ((MH "Mobile Applications") OR ( TI (App OR apps OR “mobile app*” OR “smartphone app*”) OR AB (App OR apps OR “mobile app*” OR “smartphone app*”))) **AND** ((MH "Health Behavior") OR (MH "Health Promotion") OR (MH "Exercise") OR (MH "Weight Loss") OR (MH "Obesity/DH/PC/RH/TH") OR (MH "Diet Therapy") OR (MH "Diet") OR (MH "Smoking Cessation") OR (MH "Alcohol Drinking/PC") OR (MH "Mental Health") OR (MH "Safe Sex") OR (MH "Behavioral Changes") OR (MH "Chronic Disease") OR (MH "Behavioral Objectives") OR (TI (“Health behaviour” OR “health behavior” OR “behaviour change” OR “behavior change” OR (Exercise ADJ3 (increase or start or maintain*)) OR “physical activity” OR “Weight loss” OR “healthy weight” OR “five a day” OR “diet” OR “nutrition” OR ((Maintenance OR maintain* OR achiev* Or retain*) ADJ4 (weight goal OR “weight loss” OR “goal weight” OR BMI)) OR (Smoking ADJ4 (cessation OR stop* OR quit* OR reduc*)) OR (Alcohol ADJ4 (reduc* OR limit* OR decreas* OR “cutting down” OR “cut down” OR “cut back” OR less* OR curb* OR abstain OR “dry January”)) OR “Protection from sun” OR “sun protection” OR “sun safe*” OR ((Sex OR “sex* behaviour” OR “sex* behavior”) ADJ4 (safe* OR protect*)) OR ((Alzheimer* disease OR arthritis OR asthma OR cancer OR COPD OR Crohn* disease OR cystic fibrosis OR dementia OR diabetes OR epilepsy OR heart disease OR HIV OR AIDS OR mood disorders OR bipolar OR depression OR anxiety OR multiple sclerosis OR Parkinson* disease) ADJ4 (manag* OR “self help” OR “self manag*” OR coping OR cope)) OR“Health management”) OR AB (“Health behaviour” OR “health behavior” OR “behaviour change” OR “behavior change” OR (Exercise ADJ3 (increase or start or maintain*)) OR “physical activity” OR “Weight loss” OR “healthy weight” OR “five a day” OR “diet” OR “nutrition” OR ((Maintenance OR maintain* OR achiev* Or retain*) ADJ4 (weight goal OR “weight loss” OR “goal weight” OR BMI)) OR (Smoking ADJ4 (cessation OR stop* OR quit* OR reduc*)) OR (Alcohol ADJ4 (reduc* OR limit* OR decreas* OR “cutting down” OR “cut down” OR “cut back” OR less* OR curb* OR abstain OR “dry January”)) OR “Protection from sun” OR “sun protection” OR “sun safe*” OR ((Sex OR “sex* behaviour” OR “sex* behavior”) ADJ4 (safe* OR protect*)) OR ((Alzheimer* disease OR arthritis OR asthma OR cancer OR COPD OR Crohn* disease OR cystic fibrosis OR dementia OR diabetes OR epilepsy OR heart disease OR HIV OR AIDS OR mood disorders OR bipolar OR depression OR anxiety OR multiple sclerosis OR Parkinson* disease) ADJ4 (manag* OR “self help” OR “self manag*” OR coping OR cope)) OR“Health management”))) **AND** ((MH "Outcomes (Health Care)") OR (TI (Feasibility OR usability OR “evaluat*” OR “outcome*” OR acceptability OR adherence OR “effectiv*” OR “adoption” OR “assess*”) OR AB (Feasibility OR usability OR “evaluat*” OR “outcome*” OR acceptability OR adherence OR “effectiv*” OR “adoption” OR “assess*”))) | 488 |
| Web of Science^b^ | ("cell phone" OR telemedicine OR smartphone OR "mobile phone" OR "mHealth" OR “mobile health”) **AND** (app OR apps OR "mobile app*" OR "smartphone app*") **AND** ("health behaviour" OR "health behavior" OR "behaviour change" OR "behavior change" OR (exercise NEAR/3 (increase OR start OR maintain*)) OR "physical activity" OR "weight loss" OR "exercise" OR "healthy weight" OR "five a day" OR "diet" OR nutrition OR obesity OR "nutrition therapy" OR  ((maintenance OR maintain* OR achiev* OR retain*) NEAR/4 (“weight goal” OR "weight loss" OR "goal weight" OR BMI)) OR (smoking NEAR/4 (cessation OR stop* OR quit* OR reduc*)) OR "smoking cessation" OR "smoking reduction" OR “tobacco use cessation" OR (alcohol NEAR/4 (reduc* OR limit* OR decreas* OR "cutting down" OR "cut down" OR "cut back" OR less* OR curb* OR abstain OR "dry January")) OR "protection from sun" OR "sun protection" OR "sun safe*" OR "mental health" OR "safe sex" OR ((sex OR "sex* behaviour" OR "sex* behavior") NEAR/4 (safe* OR protect*)) OR ((“Alzheimer* disease” OR arthritis OR asthma OR cancer OR COPD OR “Crohn* disease” OR “cystic fibrosis” OR dementia OR diabetes OR epilepsy OR “heart disease” OR HIV OR AIDS OR “mood disorders” OR bipolar OR depression OR anxiety OR “multiple sclerosis” OR “Parkinson* disease”) NEAR/4 (manag* OR “self help” OR “self manag*” OR coping OR cope)) OR "health management" OR "behavioral medicine" OR "chronic disease" OR "chronic condition") **AND** (outcome OR feasibility OR usability OR “evaluat*” OR “outcome*” OR acceptability OR adherence OR “effectiv*” OR “adoption” OR “assess*” ) | 1870 |

^a^MeSH terms were converted to the equivalent database-specific headings

^b^MeSH terms were included as keywords, as Web of Science does not support MeSH headings
