# Supplementary material for: Mobile Apps for Health Behavior Change in Physical Activity, Diet, Drug and Alcohol Use, and Mental Health: Systematic Review
Source: JMIR Mhealth Uhealth. 2020 Mar 18;8(3):e17046. doi: 10.2196/17046 (PMC7113799; doi:10.2196/17046)
Supplement: Multimedia Appendix 4 [file mhealth_v8i3e17046_app4.docx]

Table 4. Summary of study characteristics

| **Study (year)** | **Country** | **Study population** | **Study duration** | **Target health behaviour** | **App** |
| --- | --- | --- | --- | --- | --- |
|  |  |  |  |  |  |
| Arrogi et al. (2019) [[29]](https://paperpile.com/c/PCrjdq/nuA7) | Belgium | 51 (CC) participants (primarily office workers) | 2 weeks | Reduce sedentary behaviour | stAPP |
| Bakker et al. (2018) [[61]](https://paperpile.com/c/PCrjdq/QKXn) | Australia | 226 (CC, imput.) participants | 30 days | Mental well-being | MoodKit, MoodPrism, MoodMission |
| Baskerville et al. (2018) [[64]](https://paperpile.com/c/PCrjdq/PWkt) | Canada | 1599 ITT (725 Complete Case) young adult smokers | 6 months | Smoking cessation | Crush the Crave (CTC) |
| Bertholet et al. (2019) [[72]](https://paperpile.com/c/PCrjdq/yGsa) | Canada, US | 977 ITT (702 CC) excessive drinkers | 6 months | Reduce alcohol consumption | Alcooquizz |
| BinDhim et al. (2018) [[28]](https://paperpile.com/c/PCrjdq/ig7m) | US, UK, Australia, Singapore | 684 (ITT) daily smokers | 6 months | Smoking cessation | Quit Advisor Plus |
| Blackburne et al. (2016) [[40]](https://paperpile.com/c/PCrjdq/qpO3) | Australia | 52 (CC) participants | 2 weeks | Dietary habits | NoGo |
| Bricker et al. (2014) [[65]](https://paperpile.com/c/PCrjdq/FLZI) | US | 164 (CC) participants who smoke 5+ cigarettes daily | 8 weeks | Smoking cessation | SmartQuit, QuitGuide (control) |
| Brindal et al. (2018) [[41]](https://paperpile.com/c/PCrjdq/PrKb) | Australia | 146 (ITT) overweight and obese adults | 6 months | Dietary habits | Weight Management Program (WMP) app |
| Brindal et al. (2019) [[50]](https://paperpile.com/c/PCrjdq/gilB) | Australia | 88 ITT (61 CC) who had lost 5%+ body weight in last 2 years | 24 weeks | Dietary habits, physical activity | MotiMate |
| Buller et al. (2014) [[66]](https://paperpile.com/c/PCrjdq/5yGc) | US | 68 (CC) adult smokers | 3 months | Smoking cessation | REQ-Mobile (Real e Quit) |
| Champion et al. (2018) [[62]](https://paperpile.com/c/PCrjdq/R1Kr) | UK | 74 ITT (62 CC) adults | 30 days | Mindfulness meditation | Headspace |
| Clarke et al. (2019) [[23]](https://paperpile.com/c/PCrjdq/xF3H) | US | 149 (CC) primary household cooks + their 9-14yo child | 10 weeks | Dietary habits | VeggieBook |
| Cowdery et al. (2015) [[30]](https://paperpile.com/c/PCrjdq/UcmK) | US | 40 (ITT) adults | 12 weeks | Physical activity | Zombies, Run!, The Walk |
| Crane et al. (2018) [[27]](https://paperpile.com/c/PCrjdq/p0uv) | UK primarily | 28112 ITT (2114 CC) adult smokers who set a quit date | 3 months | Smoking cessation (manage cravings) | Smoke Free |
| Crane et al. (2018) [[67]](https://paperpile.com/c/PCrjdq/4a7S) | UK | 672 (ITT) excessive adult drinkers attempting to reduce drinking | 28 days | Reduce alcohol consumption | Drink Less |
| Direito et al. (2015) [[31]](https://paperpile.com/c/PCrjdq/dFXB) | NZ | 51 (ITT) 14-17 year olds | 8 weeks | Physical activity | Zombies, Run!, Get running (control) |
| Dunn et al. (2019) [[42]](https://paperpile.com/c/PCrjdq/x5Jj) | US | 41 (ITT) overweight and obese adults | 6 months | Dietary habits | FatSecret (calorie app), MealLogger (photo app) |
| Eyles et al. (2017) [[51]](https://paperpile.com/c/PCrjdq/rKiq) | NZ | 66 (ITT) cardiovascular disease patients | 3 months | Dietary habits, physical activity | SaltSwitch |
| Finkelstein et al. (2015) [[32]](https://paperpile.com/c/PCrjdq/LoKu) | US | 27 (CC) sedentary overweight women | 8 weeks | Reduce sedentary behaviour | Unnamed |
| Fukuoka et al. (2019) [[33]](https://paperpile.com/c/PCrjdq/s3YG) | US | 210 (ITT) inactive women | 9 months | Physical activity | mPED app |
| Gabbiadini et al. (2019) [[34]](https://paperpile.com/c/PCrjdq/Lldc) | Italy | 78 (ITT + CC) Uni of Milan students | 2 weeks | Physical activity | Pedometer or Google Fit (Android), Stepz or Pacer (iOS) |
| Garcia-Ortiz et al. (2018) [[52]](https://paperpile.com/c/PCrjdq/3ZUm) | Spain | 833 (ITT) primary care centre patients | 3 months (follow-up at 12 months) | Dietary habits, physical activity | Evident II |
| Glynn et al. (2014) [[35]](https://paperpile.com/c/PCrjdq/EYJI) | Ireland | 77 (CC) over 16 | 8 weeks | Physical activity | Accupedo-Pro Pedometer |
| Gomez-Marcos et al. (2018) [[53]](https://paperpile.com/c/PCrjdq/CFed) | Spain | 833 (ITT) primary care centre patients | 3 months (follow-up at 12 months) | Dietary habits, physical activity | Evident II |
| Gonzalez-Sanchez et al. (2019)  [[54]](https://paperpile.com/c/PCrjdq/rFz2) | Spain | 833 (ITT) primary care centre patients | 3 months (follow-up at 12 months) | Dietary habits, physical activity | Evident II |
| Harries et al. (2016) [[36]](https://paperpile.com/c/PCrjdq/Hnkq) | UK | 161 (CC) healthy males aged 18-40 | 8 weeks | Physical activity | bActive |
| Hassandra et al. (2017) [[68]](https://paperpile.com/c/PCrjdq/72xN) | Finland | 44 ITT (30 CC) smokers | 6 months | Smoking cessation (manage cravings) | Physical activity over Smoking (PhoS) |
| Hides et al. (2019) [[63]](https://paperpile.com/c/PCrjdq/JTl9) | Australia | 169 (ITT) 16-25yo with mild mental distress | 6 months | Emotional regulation | Music eScape |
| Huberty et al. (2019) [[26]](https://paperpile.com/c/PCrjdq/BhtP) | US | 88 (CC) full-time Arizona State undergrads | 12 weeks (8 weeks int.) | Mindfulness meditation | Calm |
| Hurkmans et al. (2018) [[55]](https://paperpile.com/c/PCrjdq/55V0) | Belgium | 102 (ITT) overweight adults | 12 weeks | Dietary and physical habits | b-SLIM |
| Ipjian et al. (2017) [[43]](https://paperpile.com/c/PCrjdq/PfPu) | US | 29-30 (CC) healthy adults, depending on analysis | 28 days | Dietary habits | MyFitnessPal |
| Kakoschke et al. (2018) [[44]](https://paperpile.com/c/PCrjdq/As9h) | Australia | 60 (ITT?) overweight and obese participants | 1 week (follow-up at 6 weeks) | Dietary habits | Tilt task, SEMA survey system |
| King et al. (2016) [[37]](https://paperpile.com/c/PCrjdq/FlUg) | US | 89 (CC) underactive adults >45 | 8 weeks | Physical activity | Analytic, social, and affect apps, Calorific (control) |
| Kliemann et al. (2019) [[45]](https://paperpile.com/c/PCrjdq/KNSJ) | UK | 81 (ITT) overweight and obese adults | 3 months | Dietary habits | 10 Top Tips |
| Krishnan et al. (2018) [[69]](https://paperpile.com/c/PCrjdq/iY7o) | US | 102 (ITT) adult smokers | 30 days | Smoking cessation | COach2Quit |
| Laing et al. (2014) [[46]](https://paperpile.com/c/PCrjdq/MWP6) | US | 211 (CC) overweight and obese adult primary care patients | 6 months | Dietary habits | MyFitnessPal |
| Lin et al. (2018) [[56]](https://paperpile.com/c/PCrjdq/Mm3f) | US | 310 (CC) overweight and obese (18-35yo) | 24 months | Dietary habits, physical activity | Cell Phone Intervention for You (CITY) app |
| Lopez et al. (2017) [[21]](https://paperpile.com/c/PCrjdq/n1sz) | US (Puerto Rico) | 26 (CC) overweight and obese 21-45yo | 8 weeks | Food selection, dietary habits | MyNutriCart |
| McClure et al. (2016) [[70]](https://paperpile.com/c/PCrjdq/9S0X) | US | 59-66 (CC, depending on analysis) smokers ready to quit | 5 months | Smoking cessation, medication adherence | MyMAP |
| Mummah et al. (2016) [[47]](https://paperpile.com/c/PCrjdq/jAC6) | US | 17 (ITT) overweight and obese adults | 12 weeks (of 12- month trial) | Dietary habits | Vegethon |
| Mummah et al. (2017) [[22]](https://paperpile.com/c/PCrjdq/3Cn7) | US | 135 (ITT) overweight and obese (18-50yo) | 12 months | Dietary habits | Vegethon |
| Palacios et al. (2018) [[48]](https://paperpile.com/c/PCrjdq/aZFf) | US (Puerto Rico) | 51 (CC) primary household shoppers (21-45yo) | 8 weeks | Food selection, dietary habits | MyNutriCart |
| Peiris et al. (2019) [[71]](https://paperpile.com/c/PCrjdq/LBoX) | Australia | 49 (ITT) current Aboriginal smokers >16 | 6 months | Smoking cessation | Can’t Even Quit |
| Rabbi et al. (2015) [[57]](https://paperpile.com/c/PCrjdq/v3Zh) | US | 17 (ITT) adults | 3 weeks | Dietary habits, physical activity | MyBehavior |
| Recio-Rodriguez et al. (2016) [[58]](https://paperpile.com/c/PCrjdq/4tyq) | Spain | 833 (ITT) participants | 3 months | Dietary habits (MD), physical activity | Evident II |
| Simons et al. (2018) [[38]](https://paperpile.com/c/PCrjdq/nR6P) | Belgium | 130 (ITT?) lower educated working young adults | 9 weeks (follow-up after 3 months) | Physical activity | Active Coach |
| Svetkey et al. (2015) [[59]](https://paperpile.com/c/PCrjdq/IMjl) | US | 365 (CC) overweight and obese (18-35yo) | 24 months | Dietary habits, physical activity | Cell Phone Intervention for You (CITY) app |
| van Beurden et al. (2019) [[60]](https://paperpile.com/c/PCrjdq/3CG0) | UK | 67-74 overweight and obese participants (CC, depending on analysis) | 3 months | Dietary habits, physical activity | ImpulsePal |
| Voth et al. (2016) [[39]](https://paperpile.com/c/PCrjdq/CevR) | Canada | 42 (CC) YMCA members (19-70yo) | 8 weeks | Physical activity | Unnamed |
| Walsh et al. (2016) [[24]](https://paperpile.com/c/PCrjdq/oAUE) | Ireland | 55 (CC) young adults (17-26yo) | 5 weeks | Physical activity | Accupedo-Pro Pedometer |
| Whitelock et al. (2019) [[49]](https://paperpile.com/c/PCrjdq/emXE) | UK | 107 (ITT) overweight and obese adults | 8 weeks | Dietary habits (attentive eating) | Unnamed |
| Zhou et al. (2018) [[25]](https://paperpile.com/c/PCrjdq/d2Za) | US | 64 ITT (33 CC)  UC Berkeley staff | 10 weeks | Physical activity | CalFit |
